# Population-focused community-based health literacy model for high-risk people: preventing type 2 diabetes mellitus through participatory action research in rural Thailand

**DOI:** 10.1186/s12889-026-26828-0

**Published:** 2026-03-03

**Authors:** Arunrat Utaisang, Benjayamas Pilayon, Thisachon Thanyawarathorn, Satjaporn Panomsak, Nitikorn Phoosuwan

**Affiliations:** 1 Department of Adult and Aging Nursing, Boromarajonani College of Nursing Nakhon Phanom, Nakhon Phanom University, Nakhon Phanom, Thailand; 2 Department of Community Health Nursing, Boromarajonani College of Nursing Nakhon Phanom, Nakhon Phanom University, Nakhon Phanom, Thailand; 3 Artsamat Subdistrict Health Promoting Hospital, Nakhon Phanom, Thailand; 4 Faculty of Public Health, Thammasat University, Pathum Thani, Thailand; 5 Department of Public Health and Caring Sciences, Uppsala University, Box 564, Uppsala, SE-751 22 Sweden

**Keywords:** Health literacy, Type 2 diabetes mellitus prevention, Community-based participatory research, Health promotion, Rural health, Thailand

## Abstract

**Background:**

Type 2 diabetes mellitus (T2DM) represents a significant public health challenge in Thailand, particularly in rural communities. Despite high prevalence rates, evidence-based, culturally appropriate prevention programs for high-risk populations remain limited. This study developed and evaluated a community-based health literacy model for T2DM prevention using participatory action research (PAR).

**Methods:**

We conducted a four-phase PAR study in a northeast province, Thailand, from April to October 2024. Fifteen high-risk individuals (mean age 54.73 ± 9.22 years, 86.7% female) and 37 healthcare stakeholders participated. The intervention comprised community preparation, model development, 8-week implementation with weekly group activities, and evaluation. We assessed health literacy using a validated questionnaire and measured body composition parameters. Data were analyzed using paired t-tests and Wilcoxon signed-rank tests for quantitative outcomes, and content analysis for qualitative data.

**Results:**

Health literacy scores improved significantly across all five domains from insufficient levels (mean 38.33–46.25) to good levels (mean 84.17–87.92) (*p* < 0.001). Significant improvements were observed in body age, weight, body mass index, waist circumference, visceral fat, total body fat, fat percentage, basal metabolic rate, and fasting blood glucose (all *p* < 0.05). Four key success factors emerged: community capital, healthcare system reform, self-initiated health management, and supportive monitoring without stigmatization.

**Conclusion:**

This PAR effectively improved health literacy and clinical outcomes among high-risk individuals. The model demonstrates the feasibility and effectiveness of leveraging community resources and networks for T2DM prevention in resource-limited settings. Findings support the integration of participatory approaches in primary prevention programs and offer a scalable framework for similar rural communities.

## Background

Type 2 diabetes mellitus (T2DM) represents one of the most pressing global health challenges of the 21st century, with the International Diabetes Federation estimating over 537 million adults living with diabetes worldwide in 2021 [1]. This burden disproportionately affects low- and middle-income countries (LMICs), where 80% of diabetes cases occur, often in settings with limited healthcare resources [2]. Evidence demonstrates that structured prevention programs targeting high-risk populations can reduce T2DM incidence by up to 58% and mortality rates by 73%, primarily through lifestyle modifications and early intervention [3, 4]. However, the implementation of such programs in resource-limited rural communities remains challenging, particularly in Southeast Asian contexts where cultural factors, healthcare infrastructure, and community engagement significantly influence intervention effectiveness.

Thailand exemplifies this challenge, with approximately 11% of adults living with T2DM—representing nearly six million individuals [5]. The northeastern region bears a particularly heavy burden, with Nakhon Phanom Province recording 27,560 diagnosed cases and an annual incidence rate of 10.6% [6]. This situation was further complicated by the COVID-19 pandemic, which increased both infection risk and disease severity among people with T2DM [7, 8]. Local health facilities report persistent barriers to effective diabetes prevention, including inadequate dietary control, poor medication adherence, insufficient physical activity, and stress management challenges [9]. Critically, while healthcare services focus predominantly on managing existing T2DM cases, systematic approaches to preventing disease progression among high-risk populations remain underdeveloped.

Health literacy—defined as the capacity to obtain, process, and understand basic health information needed to make appropriate health decisions—has emerged as a crucial determinant of diabetes prevention success [10, 11]. Systematic reviews indicate that enhanced health literacy enables high-risk individuals to engage more effectively in self-management behaviors, leading to improved glycemic control and reduced progression to diabetes [12, 13]. However, conventional health literacy interventions often adopt top-down approaches that fail to account for local contexts, community resources, and cultural factors that shape health behaviors in rural settings [14]. Furthermore, cross-cultural communication barriers and limited accessibility to health information frequently undermine intervention effectiveness in diverse, rural populations [15, 16].

Community-based participatory research (CBPR) offers a promising alternative framework for developing contextually appropriate health interventions [17]. By integrating community capacity, local resources, and democratic participation, CBPR approaches enable sustainable problem-solving that aligns with community priorities and leverages existing social networks [18]. This is particularly relevant in Thailand’s decentralized healthcare system, where local administrative organizations increasingly assume responsibility for primary healthcare delivery [19]. Evidence suggests that community-engaged diabetes prevention programs that mobilize local leaders, health volunteers, and social networks can achieve superior outcomes compared to facility-based interventions, particularly in rural LMICs [20, 21]. However, few studies have systematically developed and evaluated participatory health literacy models specifically designed for high-risk populations in rural northeastern Thai communities, where cultural factors, social structures, and healthcare infrastructure differ substantially from urban settings.

### Research gap and study rationale

Despite growing recognition of health literacy’s importance in prevention of diabetes, three critical gaps persist in the literature. First, most health literacy interventions employ standardized protocols developed in high-income countries, with limited adaptation to rural LMIC contexts where community structures, health beliefs, and resource availability differ substantially [22, 23]. Second, existing interventions rarely utilize genuine participatory approaches that engage communities as active co-designers rather than passive recipients of health education [24]. Third, there is insufficient evidence regarding how community-based health literacy models can be effectively integrated within decentralized healthcare systems common in Southeast Asia.

The current study addresses these gaps by developing and evaluating a health literacy model grounded in participatory action research principles and tailored to the specific context of a rural northeastern Thai community. By engaging high-risk individuals, healthcare providers, local leaders, and community volunteers as equal partners in intervention design and implementation, this feasibility study aimed to create a sustainable, culturally appropriate prevention model that can be scaled across similar settings. Understanding how community participation, local resources, and health literacy enhancement synergistically reduce T2DM risk has important implications for primary prevention strategies in resource-limited settings globally.

### Aim

This study aimed to develop and evaluate a community-based health literacy model for people at high risk for T2DM using participatory action research in a northeastern province of Thailand. Specific objectives were to: (1) identify community resources and barriers to diabetes prevention through participatory situational analysis; (2) co-design a culturally appropriate health literacy intervention with community stakeholders; (3) implement and refine the model through iterative community engagement; and (4) evaluate the model’s effectiveness in improving health literacy and clinical outcomes among high-risk individuals.

### Study design and theoretical framework

We employed a participatory action research (PAR) design integrated with community-based approaches to develop and evaluate a health literacy intervention for T2DM prevention. PAR was selected for its capacity to combine community resources with democratic participation, enabling sustainable problem-solving through iterative cycles of planning, action, observation, and reflection [13]. The intervention development was guided by Nutbeam’s health literacy framework, encompassing three progressive levels: functional health literacy (basic knowledge and skills), interactive health literacy (advanced cognitive and social skills), and critical health literacy (ability to critically analyze and use information for greater control over health determinants) [14].

**Fig. 1:**
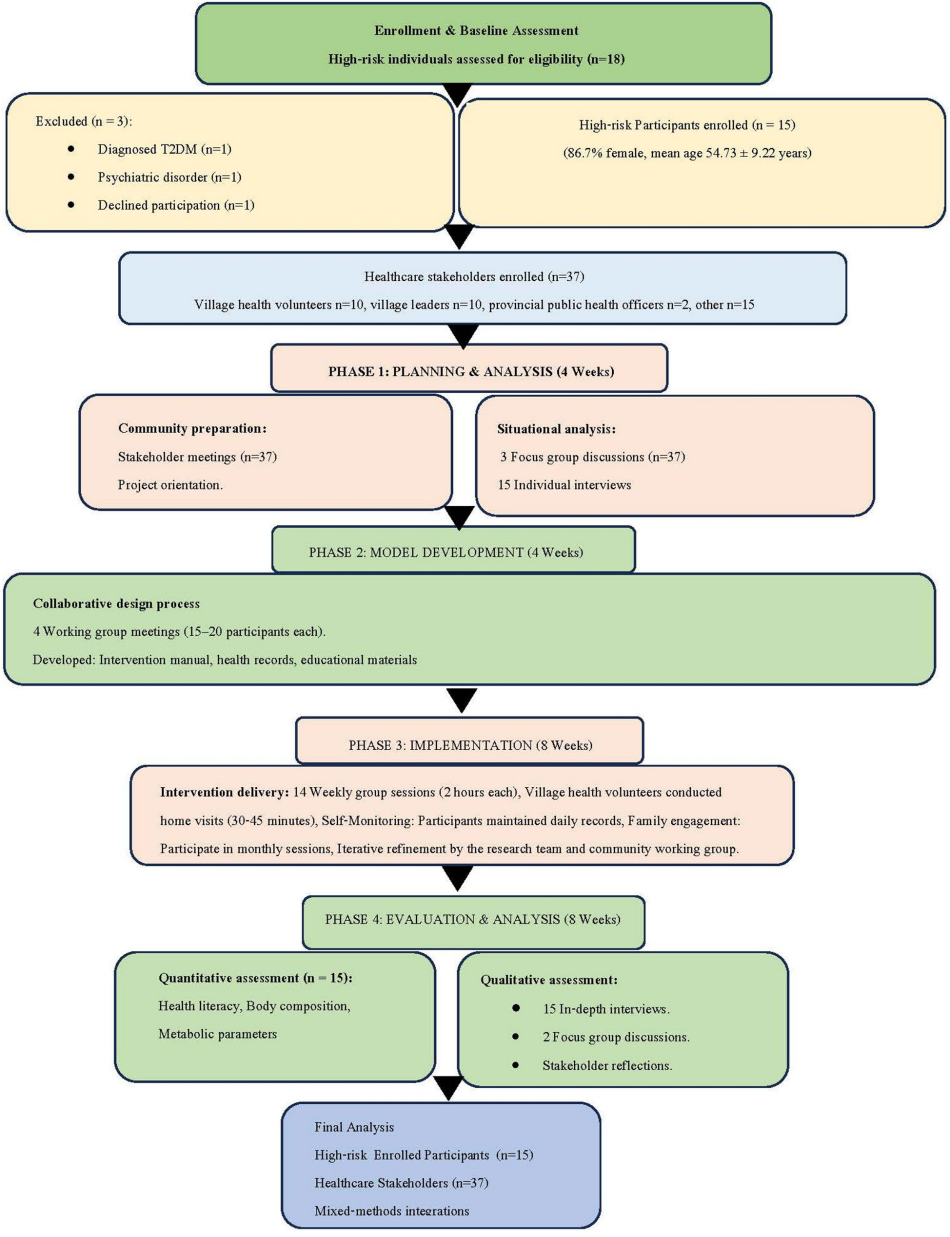
Flow diagram depicting participant recruitment, intervention phases, and assessment points in the study

**Fig. 2:**
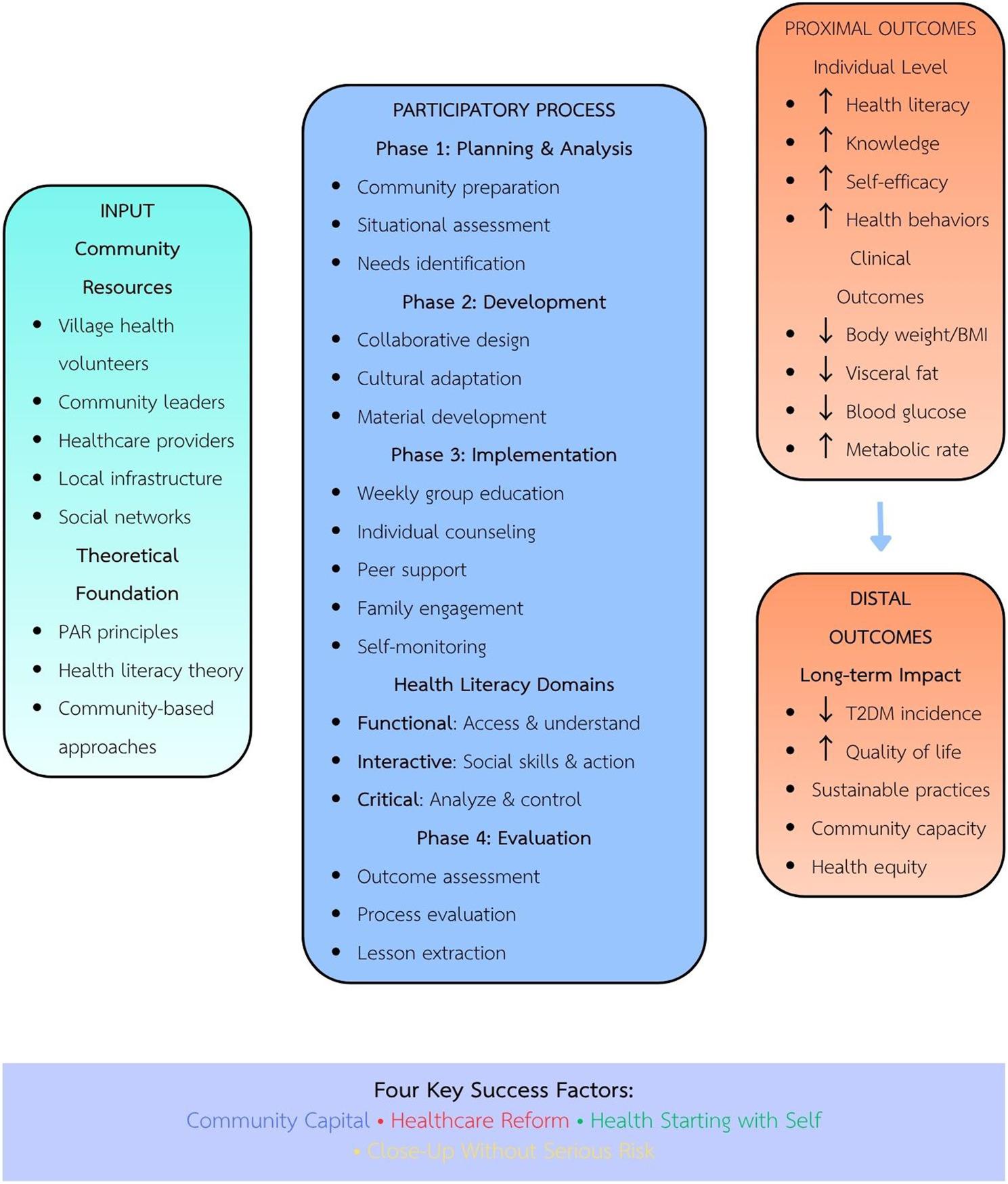
Conceptual framework for community-based health literacy intervention

The study was conducted in four sequential phases over a 24-week period from 22 April to 4 October 2024: (1) Planning and analyzing community conditions (4 weeks); (2) Development of health literacy model (4 weeks); (3) Implementation and refinement (8 weeks); and (4) Evaluation (8 weeks). This cyclical process enabled continuous feedback and adaptation based on community input and emerging findings (Figs. 1 and 2).

### Study setting

This study was conducted in Artsamart Subdistrict, Muang District, Nakhon Phanom Province, located in northeastern Thailand along the Thai-Lao border. The subdistrict comprises six villages with approximately 3,500 residents, characterized by cultural diversity (Buddhist and Christian communities) and mixed ethnicities (Thai, Laotian, Vietnamese). Healthcare services are provided through one Subdistrict Health Promotion Hospital, supported by the Provincial Administrative Organization (PAO), Subdistrict Administrative Organization (SAO), and a network of village health volunteers. The area has a T2DM prevalence of 10.6% annually, with limited systematic prevention services for high-risk populations.

### Participants

There were two distinct participant groups were recruited: Group 1 High-Risk individuals (*n* = 15) and Group 2: Healthcare stakeholders (*n* = 37).

Group 1: We recruited individuals at elevated risk for T2DM using purposive sampling. Inclusion criteria were: (1) age ≥ 18 years; (2) presence of ≥ 2 risk factors including family history of T2DM, previous COVID-19 infection, body mass index (BMI) ≥ 25 kg/m², waist circumference ≥ 90 cm (males) or ≥ 80 cm (females), or health-risk behaviors (regular consumption of sweet foods, coffee/tea, or alcohol); (3) residence in Artsamart Subdistrict; and (4) willingness to participate for the full study duration. Exclusion criteria were: (1) a diagnosis of T2DM; (2) diagnosed psychiatric disorder with uncontrolled symptoms; (3) medications affecting body weight; or (4) pregnancy.

Group 2: Healthcare stakeholders were purposively selected to ensure representation across the community health system: health promotion hospital staff (*n* = 4), village health volunteers (*n* = 10), village leaders (*n* = 10), PAO health workers (*n* = 1), SAO health workers (*n* = 5), provincial public health officers (*n* = 2), teachers (*n* = 3), nutritionist (*n* = 1), and counseling nurse (*n* = 1). All stakeholders were ≥ 18 years and willing to participate throughout the study period.

Sample size justification: The sample size for high-risk individuals was based on pragmatic considerations appropriate for PAR methodology and the subdistrict’s population. Previous community-based diabetes prevention studies in similar settings have successfully utilized samples of 10–20 participants for intensive, participatory interventions [17–19]. Given the resource-intensive nature of the weekly intervention activities and the pilot nature of model development, we determined that 15 participants would be sufficient to assess feasibility, acceptability, and preliminary effectiveness while enabling meaningful community engagement.

### Intervention development and implementation

The study consisted of four phases. A summary of the four phases is presented in Table 1.

**Table 1:** Summary of intervention and implementation program

Phase	Inputs	Activities	Outputs	Outcomes
Phase 1: Planning and analysis				
1.1 Community preparation (Weeks 1–2)	Project summary	Meetings of the community working group to define project aims and develop an action plan	Improved understanding of project objectives among community working group members	Community working group able to implement the project plan in practice
1.2 Situational analysis (Weeks 3–4)	- Guides for focus group discussion and in-depth interviews - Body composition measurements	- Conducting focus group discussion - Analysis of community data related to health literacy in type 2 diabetes	Report on community health status related to type 2 diabetes health literacy	Utilization of health data at the individual level
Phase 2: Model developments (Weeks 5–8)	- Health literacy data from situational analysis - Body composition data	- working group meetings (15–20 participants each) to collaboratively design the intervention - Consensus-building discussions to establish intervention goals, prioritize activities, define roles and responsibilities, and develop implementation protocols	- Peer support model - Individual goal-setting and progress tracking - Use of culturally appropriate communication channels	- Integration of existing community resources (exercise groups, traditional foods) - Intervention program with operational plan for health
3. Implementation and refinement (Weeks 9–16)	Implementation protocols	- Individual Support - Self-Monitoring - Family Engagement - Iterative Refinement	- Individualized plans for each participant - Improved Health literacy	- Change in body composition
4. Evaluation (Weeks 17–24)	- Health literacy Scale - Body composition measurements - Focus group discussion	Post-intervention assessments (replication of baseline measures, in-depth interviews with high-risk participants, and two FGDs with stakeholders.	- Changes in body composition - Lessons learned reported by participants - Lessons learned reported by the working group	Finalized implementation model

### Phase 1: Planning and analysis (Weeks 1–4)

#### Community preparation (Week 1–2)

We initiated the project through a stakeholder meeting involving all 37 healthcare stakeholders. The research team presented the project aims, PAR principles, participation methods, and expected outcomes. A 90-minute facilitated discussion explored current diabetes prevention challenges, community resources, and potential solutions.

#### Situational analysis (Week 3–4)

We conducted three focus group discussions (FGDs) with stakeholders (10–15 participants each, 90–120 min) to analyze community conditions, identify strengths and barriers, and envision future improvements. FGDs were audio-recorded, transcribed verbatim, and analyzed using inductive content analysis to identify key themes. Concurrently, individual baseline assessments were conducted with high-risk participants, including health literacy evaluation, body composition measurement, and in-depth interviews exploring T2DM knowledge, perceived risks, and self-care practices.

### Phase 2: Model development (Weeks 5–8)

Based on Phase 1 findings, we convened four working group meetings (15–20 participants each) to collaboratively design the intervention. Participants included high-risk individuals, health volunteers, healthcare providers, and local leaders. Through iterative discussion and consensus-building, the group established intervention goals, prioritized activities, defined roles and responsibilities, and developed implementation protocols. Key decisions included:

- Weekly group meetings at the village temple (neutral, accessible location).
- Peer support model with village health volunteers as facilitators.
- Individual goal-setting and progress tracking using self-monitoring tools.
- Integration of existing community resources (exercise groups, traditional foods).
- Use of culturally appropriate communication channels (LINE groups, home visits).

The working group developed intervention materials including: (1) self-care management manual; (2) health record booklet for tracking diet, physical activity, and body composition; (3) educational materials on T2DM risk factors and prevention strategies; and (4) facilitator guide for village health volunteers.

### Phase 3: Implementation and refinement (Weeks 9–16)

The intervention was implemented over an 8-week period, incorporating the following components:

#### Weekly group sessions (2 h)

Participants met weekly at the village temple, facilitated by trained village health volunteers and supervised by healthcare providers. Sessions included: health education using interactive methods (discussions, demonstrations, role-plays) delivered by community healthcare providers; goal-setting and action planning for dietary modification and physical activity conducted by a nutritionist at a community hospital; peer support and experience sharing facilitated by trained village health volunteers; barrier problem-solving; and progress review using individual health records by healthcare providers, with feedback provided by a registered nurse to support participants’ health improvement. In addition, a psychologist and an exercise trainer were involved upon participants’ request. Topics rotated weekly, covering nutrition principles, portion control, physical activity, stress management, blood glucose monitoring, and complications prevention. 

#### Individual support

Village health volunteers conducted home visits (30–45 min) fortnightly to provide personalized guidance, assess progress, address barriers, and reinforce learning. A LINE messaging group enabled real-time support, question-answering, and peer encouragement between sessions. 

#### Self-monitoring

Participants maintained daily records of food intake, physical activity duration, body weight, and blood glucose (when available) using the standardized health record booklet. Records were reviewed weekly during group sessions by the healthcare providers. An example of daily records included in the booklet is presented in Table 2.

**Table 2:** Example of daily records included in the booklet

Contents	Example
Food record for each meal	
Exercise plan for everyday life	

#### Family Engagement

Family members were invited to participate in four sessions conducted monthly to increase household support for dietary and lifestyle changes. 

#### Iterative refinement

The research team and community working group met biweekly for one hour to review implementation fidelity, discuss challenges, and adapt activities based on participant feedback. Modifications included adjusting session timing for agricultural schedules, incorporating seasonal local foods into dietary guidance, and adding group physical activities (walking, traditional dance).

### Phase 4: Evaluation (Weeks 17–24)

Post-intervention assessments replicated baseline measures at Week 24 (two months after the final session). We conducted in-depth interviews with all high-risk participants (30–45 min each) and two FGDs with stakeholders (90 min each) to explore intervention experiences, perceived outcomes, sustainability factors, and recommendations for model refinement.

### Measurement

#### Quantitative instruments

Participant information form: We collected sociodemographic data (age, gender, education, occupation), medical history (chronic diseases, family diabetes history, COVID-19 infection), and health behaviors (smoking, alcohol consumption, dietary patterns, physical activity, sleep duration).

Health literacy assessment tool: We used a validated 20-item questionnaire developed by Kaewdumkoeng (2022) assessing five health literacy domains: access (four items), understanding (4 items), appraisal (4 items), application (four items), and communication (four items) [14]. Items used a 5-point Likert scale (1 = never to 5 = always). Total scores ranged from 20 to 100, categorized as insufficient (< 60), moderate (60–79), or good (≥ 80). The instrument demonstrated excellent internal consistency (Cronbach’s α = 0.917) in our pilot testing.

Body composition assessment: We measured height (stadiometer, nearest 0.1 cm), weight (digital scale, nearest 0.1 kg), waist circumference (measuring tape at midpoint between lowest rib iliac crest, nearest 0.1 cm), and comprehensive body composition using bioelectrical impedance analysis (body fat percentage, visceral fat level, muscle mass, basal metabolic rate). BMI was calculated as weight(kg)/height(m²). Fasting capillary blood glucose was measured using a glucometer (Model VGM03) following an overnight fast of at least 8 h and abstinence from alcohol. All electronic devices, including the glucometer, were calibrated annually by the provincial hospital. Body composition was assessed using a single measurement between 6:00 and 8:00 a.m. on the scheduled date. All measurements were conducted by trained research assistants using standardized protocols at baseline and at post-intervention (Week 24). 

#### Qualitative instruments

FGD guide: We developed a semi-structured guide exploring: current diabetes prevention practices, community resources and barriers, perceptions of health literacy needs, ideal characteristics of prevention programs, and suggestions for community engagement strategies. Example questions: “What challenges do community members face in preventing diabetes?” “What community resources could support diabetes prevention?” “How can we develop health services that communities will actually use?”

In-depth interview guide: We created a guide for individual interviews with high-risk participants covering: T2DM knowledge and understanding, perceived personal risk, current self-care practices, barriers to healthy behaviors, support needs, and intervention experiences (post-intervention only).

Self-care management manual and health record: Participants received a comprehensive manual including: T2DM information, healthy eating guidelines adapted to local cuisine, physical activity recommendations, stress management techniques, and self-monitoring instructions. The accompanying health record included a daily food diary, physical activity log, body weight tracker, blood glucose record (for participants who chose to have their levels assessed at the health-promoting hospital), and reflection space for goal progress. To minimize bias arising from self-reported data and recall, participants were instructed to record their data daily or as soon as possible. They were informed that their responses would be kept confidential and anonymous, used solely for research purposes, and would not affect the healthcare services they received from the hospital. 

#### Instrument quality assurance

Three experts in community health, diabetes prevention, and health literacy reviewed all instruments for content validity. Item-level content validity indices ranged from 0.80 to 1.0, indicating excellent content validity. We pilot-tested quantitative instruments with 30 individuals from a neighboring subdistrict with similar characteristics. Internal consistency was excellent for health literacy assessment (Cronbach’s α = 0.917) and self-care management items (Cronbach’s α = 0.925).

Qualitative instruments were pilot-tested with two participants per group to ensure clarity and cultural appropriateness. We established credibility using triangulation (multiple data sources, methods, and investigators), member checking (participants reviewed preliminary findings), and peer debriefing (regular team discussions) [15, 16].

### Data analysis

Quantitative analysis: Data were analyzed using IBM SPSS Statistics version 26.0. We calculated descriptive statistics (frequencies, percentages, means, standard deviations) for participant characteristics and baseline variables. We assessed normality using the Shapiro-Wilk test and visual inspection of histograms and Q-Q plots.

For normally distributed continuous variables (body age, weight, BMI, waist circumference, visceral fat, total body fat, fat percentage, basal metabolic rate, fasting glucose), we used paired-samples t-tests to compare pre- and post-intervention values. For non-normally distributed variables (muscle mass, health literacy scores), we used Wilcoxon signed-rank tests. Statistical significance was set at *p* < 0.05 (two-tailed). We calculated effect sizes using Cohen’s d for t-tests and r for Wilcoxon tests to assess practical significance.

Qualitative analysis: Audio-recorded FGDs and interviews were transcribed verbatim in Thai. Two researchers independently conducted inductive content analysis using the following steps: (1) familiarization through repeated reading; (2) generating initial codes; (3) organizing codes into categories; (4) developing themes; (5) reviewing and refining themes; and (6) defining and naming final themes [16]. Researchers met regularly to discuss coding decisions, resolve discrepancies through consensus, and ensure analytical rigor. We used NVivo 12 software to manage qualitative data. Representative quotations were translated to English for reporting, with back-translation performed to ensure accuracy.

### Integration of findings

Following a convergent mixed-methods design, we integrated quantitative and qualitative findings during interpretation. Qualitative themes were used to explain and contextualize quantitative outcomes, while quantitative results provided evidence of intervention effects identified in qualitative data. The four success factors emerged through integration of both data types.

### Ethical considerations

This study received ethical approval from Nakhon Phanom University Ethics Committee (Order 102/2567, No.10267, 4 April 2024) and was conducted according to the Declaration of Helsinki principles. We obtained written informed consent from all participants after providing comprehensive oral and written information in Thai about study purposes, procedures, risks, benefits, voluntary participation, and withdrawal rights. Participants received no monetary compensation but were provided free health screenings, educational materials, and ongoing support.

We protected participant confidentiality by: assigning identification codes, storing data in password-protected files, conducting interviews in private spaces, and reporting only aggregate or anonymized data. Village health volunteers completed research ethics training emphasizing confidentiality and respect for participant autonomy. Participants demonstrating abnormal glucose levels or other health concerns were referred to appropriate healthcare services.

## Results

### Participant characteristics

Fifteen individuals at high risk for T2DM participated in the study. The majority were female (86.7%, *n* = 13), with ages ranging from 43 to 76 years (M = 54.73, SD = 9.22). The most common occupation was homemaker (40.0%, *n* = 6), followed by employee (20.0%, *n* = 3). Most participants (86.7%, *n* = 13) reported no chronic diseases, while 13.3% (*n* = 2) had hypertension. Two participants (13.3%) were current smokers, and four (26.7%) consumed alcohol. All participants (100%, *n* = 15) regularly consumed spicy foods and drank coffee, while 73.3% (*n* = 11) consumed soda. Regarding dietary patterns, 60.0% (*n* = 9) reported consuming more than three portions of vegetables daily, and 93.3% (*n* = 14) consumed more than two portions of fruit daily. However, 86.7% (*n* = 13) reported not engaging in regular physical exercise. Most participants (93.3%, *n* = 14) reported sleeping at least seven hours per night.

Thirty-seven healthcare stakeholders participated throughout the four study phases. The stakeholder group comprised health promotion hospital staff (*n* = 4), village health volunteers (*n* = 10), village leaders (*n* = 10), PAO) health worker (*n* = 1), SAO health workers (*n* = 5), provincial public health officers (*n* = 2), teachers (*n* = 3), nutritionist (*n* = 1), and counseling nurse (*n* = 1). See Table 3.

**Table 3:** Sociodemographic and health characteristics of high-risk participants (*n* = 15)

Characteristic	*n* (%) or M ± SD
**Sociodemographic**	
Age (years)	54.73 ± 9.22
Age range (years)	43–76
Gender	
Female	13 (86.7)
Male	2 (13.3)
Occupation	
Homemaker	6 (40.0)
Employee	3 (20.0)
Farmer	3 (20.0)
Self-employed	2 (13.3)
Other	1 (6.7)
**Medical history**	
Chronic diseases	
None	13 (86.7)
Hypertension	2 (13.3)
Family history of T2DM	15 (100.0)
Previous COVID-19 infection	15 (100.0)
**Health Behaviors**	
Current smoker	2 (13.3)
Alcohol consumption	4 (26.7)
Regular coffee consumption	15 (100.0)
Soda consumption	11 (73.3)
Spicy food consumption	15 (100.0)
Vegetable intake (> 3 portions/day)	9 (60.0)
Fruit intake (> 2 portions/day)	14 (93.3)
Regular physical exercise (≥ 3 times/week)	2 (13.3)
Adequate sleep (≥ 7 h/night)	14 (93.3)
**Baseline clinical measurements**	
Body mass index (kg/m²)	26.84 ± 3.45
Normal weight (18.5–22.9)	3 (20.0)
Overweight (23.0-24.9)	4 (26.7)
Obese (≥ 25.0)	8 (53.3)
Waist circumference (cm)	88.73 ± 8.92
High risk (≥ 90 cm male, ≥ 80 cm female)	11 (73.3)
Fasting blood glucose (mg/dL)	105.27 ± 12.54
Normal (< 100)	4 (26.7)
Prediabetes (100–125)	11 (73.3)

Values are presented as mean ± standard deviation or frequency (percentage). Asian BMI cutoffs used: normal weight 18.5–22.9, overweight 23.0-24.9, obese ≥ 25.0 kg/m²

*M *Mean, *SD *Standard Deviation, *T2DM *Type 2 Diabetes Mellitus

### Phase 1: Community preparation and situational analysis

#### Community stakeholder perspectives

Three FGDs with healthcare stakeholders (*n* = 37) revealed four primary themes regarding diabetes prevention challenges and opportunities in the community.

Theme 1: Inadequate prevention infrastructure

Stakeholders identified that while the community had substantial resources for diabetes management, systematic approaches to prevention were lacking.

“We have good systems for taking care of people who already have diabetes, but we don’t have a clear way to help people who are at risk before they develop the disease.” (Village Health Volunteer 03).

Theme 2: Need for community-centered approaches

Healthcare providers expressed frustration with top-down approaches that failed to engage community members as active participants.

“When we just tell people what to do, they don’t follow through. But when we work together to find solutions that fit their lives, they’re more committed.” (Health Promotion Hospital Staff 02).

Theme 3: Untapped community resources

Stakeholders recognized existing community assets that could support prevention efforts, including village temples as neutral gathering spaces, strong social networks among villagers, respected community leaders, and seasonal agricultural calendars that could inform activity timing.

Theme 4: Cultural barriers to behavior Change

Participants identified cultural factors influencing health behaviors, including traditional food preferences, social obligations around food sharing, and stigma associated with being “sick” or “at risk”.

#### High-risk individual perspectives

In-depth interviews with high-risk individuals (*n* = 15) revealed limited awareness of personal diabetes risk, uncertainty about prevention strategies, and desire for practical, accessible guidance. Most participants (12/15) could not accurately describe their personal risk factors despite having multiple risk indicators. Several expressed that receiving a formal “at-risk” designation motivated them to participate.

“When the health volunteer told me I was at high risk, I was shocked. I thought I was healthy. Now I want to know what I can do to prevent diabetes.” (Participant 08).

### Phase 2: Collaborative intervention development

Based on Phase 1 findings, the community working group collaboratively designed the intervention over four structured meetings (15–20 participants each). Key decisions reached through consensus included:

Intervention location and timing

- Weekly meetings at the village temple (accessible, neutral, familiar location)
- Tuesday evenings (6:00–8:00 PM) to accommodate agricultural schedules
- 14-week duration balancing feasibility and adequate exposure

Facilitation model

- Village health volunteers as primary facilitators (trusted, culturally knowledgeable)
- Healthcare provider supervision and technical support
- Peer support emphasis through group activities

Content and activities

- Interactive educational sessions on diabetes risk factors, nutrition, physical activity, and stress management
- Individual goal-setting and action planning
- Self-monitoring using health record booklets
- Family engagement through monthly sessions

Support mechanisms

- Biweekly home visits by village health volunteers
- LINE messaging group for ongoing support and questions
- Integration with existing community activities (e.g., morning exercise groups)

Cultural adaptations

- Dietary guidance featuring local, seasonal foods
- Respect for food-sharing traditions while promoting portion control
- Non-stigmatizing language avoiding labels like “sick” or “patient”
- Integration of Buddhist temple and community rituals

The working group developed a comprehensive intervention manual, participant health record booklet, educational materials, and facilitator guide. All materials underwent community review and refinement before implementation.

### Phase 3: Intervention implementation and participant engagement

#### Implementation fidelity

All 14 planned weekly group sessions were conducted as scheduled. Attendance rates were high, with participants attending an average of 12.4 sessions (88.6%, range: 10–14). Village health volunteers completed 95.2% of planned home visits (200/210 total visits). The LINE messaging group maintained active engagement, with participants posting an average of 3.2 messages per week (range: 1–8) and healthcare providers responding to queries within 24 h in 96.4% of cases.

#### Intervention adaptations

Three significant adaptations were made during implementation based on participant feedback and community working group meetings:

1. Session timing adjustment (Week 3): Changed from 90 min to 120 min to allow more peer discussion time.
2. Physical activity modification (Week 5): Added group walking before sessions and traditional dance activities to accommodate varying fitness levels.
3. Dietary guidance refinement (Week 7): Incorporated seasonal fruit guidance addressing mango season overconsumption concerns.

#### Participant self-monitoring

Participants maintained daily health records with varying consistency. Adherence to self-monitoring was highest for body weight tracking (93.3% of participants recorded ≥ 5 days/week), followed by food intake documentation (80.0%), physical activity logging (73.3%), and blood glucose recording when meters were available (46.7%).

### Phase 4: Evaluation outcomes

#### Health literacy changes

Health literacy scores improved significantly across all five domains from pre- to post-intervention. Mean scores increased from insufficient levels at baseline (range: 38.33–46.25) to good post-intervention levels (range: 84.17–87.92). All improvements were statistically significant at *p* < 0.001. Effect sizes were large, ranging from *r* = 0.87 to *r* = 0.91 across domains, indicating substantial practical significance.

The greatest absolute improvement occurred in the communication domain (mean difference: 45.84 points), followed by application (45.00 points) and appraisal (44.58 points). The access domain showed the smallest but still substantial improvement (41.67 points) See Table 4.

**Table 4:** Changes in health literacy scores by domains (*n* = 15)

Domain	Pre-intervention M ± SD	Post-intervention M ± SD	Mean Difference	95% CI	Z	*p*-value	Effect Size(*r*)
Access	46.25 ± 8.34	87.92 ± 6.15	41.67	37.82, 45.52	-3.41	< 0.001	0.88
Understanding	42.50 ± 9.12	86.67 ± 5.83	44.17	39.95, 48.39	-3.42	< 0.001	0.88
Appraisal	38.33 ± 10.45	84.17 ± 7.21	45.84	40.87, 50.81	-3.41	< 0.001	0.88
Application	40.83 ± 11.23	85.83 ± 6.44	45.00	39.45, 50.55	-3.42	< 0.001	0.88
Communication	39.17 ± 12.08	85.00 ± 6.89	45.83	[39.67, 51.99]	-3.41	< 0.001	0.88
Total Score	207.08 ± 48.22	429.59 ± 30.52	222.51	[198.26, 246.76]	-3.41	< 0.001	0.88

Health literacy scores range from 20–100 per domain (total 100–500). Scores categorized as: insufficient (< 60), moderate (60–79), good (≥ 80). Wilcoxon signed-rank test was used due to non-normal distribution. Effect size (r) calculated as Z/√(2 N). All effect sizes are large (*r* > 0.5)

*M *Mean, *SD* Standard Deviation, *CI *Confidence Interval

### Body composition and metabolic outcomes

Significant improvements were observed in multiple anthropometric and metabolic parameters. Participants demonstrated statistically significant reductions in:

- Body age: decreased by 2.93 years (t = 4.80, *p* < 0.001, Cohen’s d = 1.24)
- Body weight: decreased by 2.79 kg (t = 7.13, *p* < 0.001, Cohen’s d = 1.84)
- Body mass index: decreased by 1.08 kg/m² (t = 7.42, *p* < 0.001, Cohen’s d = 1.92)
- Waist circumference: decreased by 3.35 cm (t = 4.70, *p* < 0.001, Cohen’s d = 1.21)
- Visceral fat level: decreased by 0.87 units (t = 3.89, *p* = 0.002, Cohen’s d = 1.00)
- Total body fat: decreased by 1.95 kg (t = 4.13, *p* = 0.001, Cohen’s d = 1.07)
- Body fat percentage: decreased by 1.27% (t = 2.36, *p* = 0.033, Cohen’s d = 0.61)
- Basal metabolic rate: increased by 32.53 kcal/day (t = 5.21, *p* < 0.001, Cohen’s d = 1.35)
- Fasting blood glucose: decreased by 6.47 mg/dL (t = 2.36, *p* = 0.009, Cohen’s d = 0.61)

All effect sizes were medium to large, indicating clinically meaningful changes. No significant change was observed in muscle mass (Z=-1.42, *p* = 0.15). See Table 5.

**Table 5:** Changes in body composition and metabolic parameters (*n* = 15)

Parameter	Pre-intervention M ± SD	Post-intervention M ± SD	Mean Difference	95% CI	t or Z	*p*-value	Effect Size (d or *r*)
Anthropometric Measures							
Body age (years)ᵃ	57.67 ± 9.45	54.73 ± 9.22	-2.93	-4.18, -1.69	4.80ᵗ	< 0.001	1.24ᵈ
Weight (kg)ᵃ	67.15 ± 11.23	64.36 ± 10.87	-2.79	-3.61, -1.97	7.13ᵗ	< 0.001	1.84ᵈ
Body mass index (kg/m²)ᵃ	26.84 ± 3.45	25.76 ± 3.28	-1.08	-1.39, -0.77	7.42ᵗ	< 0.001	1.92ᵈ
Waist circumference (cm)ᵃ	88.73 ± 8.92	85.38 ± 8.54	-3.35	-4.90, -1.80	4.70ᵗ	< 0.001	1.21ᵈ
Body Composition							
Visceral fat level (units)ᵃ	10.47 ± 3.18	9.60 ± 2.97	-0.87	-1.36, -0.38	3.89ᵗ	0.002	1.00ᵈ
Total body fat (kg)ᵃ	22.85 ± 6.34	20.90 ± 5.89	-1.95	-2.97, -0.93	4.13ᵗ	0.001	1.07ᵈ
Body fat percentage (%)ᵃ	33.47 ± 7.21	32.20 ± 6.98	-1.27	-2.41, -0.13	2.36ᵗ	0.033	0.61ᵈ
Muscle mass (kg)ᵇ	21.30 ± 3.82	21.46 ± 3.74	0.16	-0.08, 0.40	-1.42ᶻ	0.155	0.26ʳ
Metabolic Parameters							
Basal metabolic rate (kcal/day)ᵃ	1,284.53 ± 156.78	1,317.06 ± 152.34	32.53	19.82, 45.24	5.21ᵗ	< 0.001	1.35ᵈ
Fasting blood glucose (mg/dL)ᵃ	105.27 ± 12.54	98.80 ± 10.32	-6.47	-11.13, -1.81	2.36ᵗ	0.009	0.61ᵈ

Effect size interpretation: 13 of 14 parameters showed statistically significant improvements with medium to large effect sizes, indicating clinically meaningful changes. Muscle mass showed no significant change, likely due to the 14-week intervention duration being insufficient for muscle mass gains

*M *Mean, *SD *Standard Deviation, *CI *Confidence Interval, *t *paired t-test statistic, *Z *Wilcoxon signed-rank test statistic, *d *Cohen’s d effect size, *r *effect size for non-parametric tests (Z/√2 N)

ᵃ Analyzed using paired-samples t-test (normally distributed)

ᵇ Analyzed using Wilcoxon signed-rank test (non-normally distributed)

ᵗ t-statistic (df = 14)

ᶻ Z-statistic from Wilcoxon signed-rank test

ᵈ Cohen’s d effect size: small (0.2), medium (0.5), large (0.8)

ʳ Effect size r: small (0.1), medium (0.3), large (0.5)

#### Individual clinical outcomes

At baseline, 73.3% (*n* = 11) of participants had fasting glucose levels in the prediabetes range (100–125 mg/dL), while 26.7% (*n* = 4) had normal fasting glucose. Post-intervention, 60.0% (*n* = 9) achieved normal fasting glucose levels, 33.3% (*n* = 5) remained in the prediabetes range with reduced values, and one participant (6.7%) progressed from borderline to prediabetes despite overall group improvements. This individual was referred for intensive follow-up. 

#### Qualitative outcomes: key success factors

Integration of qualitative data from post-intervention interviews and FGDs revealed four interconnected factors contributing to intervention success.

Success Factor 1 (Community Capital): Participants and stakeholders emphasized how existing community resources enabled intervention implementation and sustainability. The subdistrict possessed valuable assets including physical infrastructure (temple, community hall), human resources (trained volunteers, engaged leaders), and social capital (trust networks, collective efficacy).

“If we would like to improve our community, the people in the community need to help each other rather than have just one person do everything. Our community actually has enough capital like schools, sports grounds, and strong leaders. We just needed to bring everything together for diabetes prevention.” (Village Leader 03)

Village health volunteers described how their established relationships and cultural knowledge enhanced intervention credibility and reach.

“People trust us because we’re their neighbors. We speak the same language, eat the same foods, face the same challenges. When we talk about preventing diabetes, they listen because they know we understand their lives.” (Village Health Volunteer 07)

Success Factor 2 (Healthcare reform): Healthcare providers and administrators recognized that effective prevention requires shifting from provider-centered clinical care to community-centered health promotion. This reorientation aligned with Thailand’s primary healthcare strengthening initiatives.

“Providing health services nowadays involves too many workloads for hospitals. We need to do projects, follow policies, and yield results instead of providing incomplete comprehensive care. If we have an option to enable people to take care of themselves with community support, it creates sustainability and frees us to provide clinical care where it’s needed.” (Health Promotion Hospital Director)

SAO health workers emphasized that integrating prevention into community structures rather than creating parallel systems reduced resource requirements and improved sustainability.

“Having people take care of themselves with the community involved and health networks assisting in monitoring creates a system that can continue after the research ends. We’re not creating something new—we’re strengthening what already exists.” (SAO Health Worker 02)

Success Factor 3 (Health starting with self): Participants internalized the concept that health management is primarily an individual responsibility supported by community resources, rather than solely a healthcare provider responsibility. This shift reflected development of critical health literacy and health autonomy.

“Our body, we should take care of ourselves. If we do not take care of ourselves, who will do it? I used to think doctors and nurses should tell me what to do. Now I understand that good health is not for sale—I have to make it myself with knowledge and support from my community.” (Participant 15)

Several participants described increased confidence in making health decisions and problem-solving barriers independently.

“Before, when I faced a problem like wanting to eat sweets, I would just eat them and feel guilty. Now I think about why I want sweets, what else could satisfy me, and how to balance enjoyment with health. I make my own decisions based on what I learned.” (Participant 06)

Success Factor 4 (Close-up without serious risk): Participants valued supportive monitoring that avoided stigmatization, medicalization, or alarmism. The “at-risk” framing motivated behavior change without inducing excessive anxiety or helplessness. Regular check-ins and feedback demonstrated care and accountability while respecting autonomy.

“The health volunteers check on me and encourage me, but they don’t scold me or make me feel like I’m sick or failing. They help me understand my progress and think about next steps. This makes me want to continue rather than give up or avoid them.” (Participant 11)

Healthcare providers noted that non-judgmental support facilitated engagement among individuals who might avoid traditional healthcare settings due to fear of criticism.

“When we work in the community instead of the clinic, and focus on support instead of authority, people are more open. They share their challenges, ask questions, and stay engaged. Fear and shame are barriers to prevention—removing them is essential.” (Counseling Nurse)

The LINE messaging group exemplified accessible, non-threatening support.

“Nowadays, there are convenient communication channels to provide greater access to health services. We created LINE groups where people at risk can support each other and get expert advice when needed. People ask questions they might be embarrassed to ask face-to-face.” (Provincial Public Health Officer 01)

#### Synthesis of mixed-methods findings

Integration of quantitative and qualitative findings revealed complementary insights. Quantitative data demonstrated significant improvements in health literacy and clinical outcomes, while qualitative data illuminated mechanisms and contextual factors explaining these improvements. The four success factors identified qualitatively—community capital, healthcare reform, self-initiated health management, and supportive monitoring—provided theoretical and practical understanding of how the participatory intervention produced observed outcomes.

Participants who articulated stronger internalization of “health starting with self” (critical health literacy) in interviews tended to demonstrate greater improvements in body composition parameters, suggesting that critical health literacy may mediate between knowledge acquisition and sustained behavior change. Similarly, participants who actively engaged in the LINE group and peer support showed higher intervention attendance and self-monitoring adherence, indicating that social support mechanisms facilitated program engagement and outcomes.

The convergence of quantitative effectiveness evidence and qualitative mechanistic insights strengthens confidence in both the intervention’s impact and the validity of the community-based participatory approach. Findings suggest that genuine community participation not only enhances intervention relevance and sustainability but may also amplify effectiveness through mechanisms including enhanced trust, cultural resonance, peer accountability, and community ownership.

## Discussion

This PAR demonstrated that a community-based health literacy model significantly improved both health literacy and clinical outcomes among individuals at high risk for T2DM in rural northeastern Thailand. Health literacy scores across all five domains increased substantially from insufficient to good levels (pre-intervention: 38.33–46.25; post-intervention: 84.17–87.92; *p* < 0.001), representing large effect sizes. Concurrently, participants achieved significant improvements in multiple anthropometric and metabolic parameters, including body weight, BMI, waist circumference, visceral fat, body fat percentage, basal metabolic rate, and fasting blood glucose (all *p* < 0.05). Qualitative findings revealed four key success factors underpinning intervention effectiveness: leveraging community capital, reforming healthcare delivery approaches, promoting self-initiated health management, and providing supportive monitoring without stigmatization. These findings suggest that participatory, community-engaged approaches can effectively enhance preventive capacity among high-risk populations in resource-limited settings.

### Health literacy enhancement

Our findings align with accumulating evidence that structured health literacy interventions can improve self-management capabilities and clinical outcomes among individuals at risk for T2DM [17–19]. Prathangam’s community model in northeastern Thailand similarly reported improved health behaviors using the 3A2S principles, though that study focused on behavior change rather than comprehensive health literacy development [17]. Najarus et al. documented comparable health literacy improvements among individuals with impaired fasting glucose in northern Thailand, achieving blood glucose control through educational programming [19]. Our study extends this evidence by demonstrating effectiveness specifically in high-risk populations before glucose dysregulation develops, emphasizing primary prevention rather than progression delay. In addition, participants were given the opportunity to self-assess their body composition, which may have contributed to improvements in their health literacy.

Internationally, systematic reviews confirm that diabetes self-management education improves glycemic control and quality of life among individuals with established T2DM [20]. However, fewer studies have examined health literacy interventions for primary prevention in community settings, particularly using participatory methodologies. Our integration of functional, interactive, and critical health literacy dimensions within a participatory framework addresses this gap and demonstrates feasibility in rural LMIC contexts where healthcare resources are constrained.

### Body composition and metabolic outcomes

The significant improvements in body composition parameters observed in our study are consistent with lifestyle modification trials demonstrating that weight reduction, increased physical activity, and dietary changes can prevent or delay T2DM onset among high-risk individuals [25, 26]. The magnitude of changes we observed—though modest in absolute terms—are clinically meaningful for diabetes risk reduction. Even modest weight loss (5–7% of body weight) and visceral fat reduction substantially decrease T2DM incidence in high-risk populations [25]. Our intervention achieved these changes through low-cost, community-implemented strategies rather than resource-intensive clinical programs, suggesting potential for sustainable, scalable prevention. A key contributor to this improvement was participants’ self-monitoring, which involved recording their behaviors and consulting experts of their choice, allowing them to track their progress and independently enhance their health practices.

The absence of significant muscle mass changes warrants discussion. Sarcopenic obesity—characterized by increased adiposity concurrent with reduced muscle mass—develops gradually, particularly after age 30, with muscle loss accelerating through inactivity [21, 22]. Our 14-week intervention may have been insufficient to demonstrate measurable muscle mass gains, especially given participants’ mean age of 54.73 years and baseline low physical activity levels. Longer intervention durations incorporating progressive resistance training may be necessary to achieve muscle mass preservation or enhancement, which would further improve metabolic health and diabetes prevention [22]. 

### Community-based participatory approaches

Our findings strongly support the effectiveness of community-based participatory approaches for diabetes prevention, corroborating systematic review evidence from LMICs [23]. A meta-analysis study found that community-based programs in LMICs significantly reduced diabetes incidence and improved metabolic parameters, with effect sizes comparable to those observed in our study [23]. Similarly, a qualitative study in Thai communities identified that effective diabetes risk management requires shared responsibility among individuals, families, healthcare providers, and community leaders—precisely the multi-stakeholder engagement we achieved through PAR [24].

The four success factors emerging from our qualitative analysis provide important insights into mechanisms underlying intervention effectiveness. Community capital—including existing social networks, trusted leaders, and physical infrastructure—enabled intervention implementation without substantial external resources. Healthcare reform thinking, particularly the shift from provider-centered to community-centered prevention, aligned with Thailand’s decentralization policy and enabled sustainable integration within existing systems [12]. Self-initiated health management reflected participants’ internalization of critical health literacy, moving beyond knowledge acquisition to autonomous decision-making and behavior change [27]. Finally, supportive monitoring without stigmatization addressed a critical barrier in diabetes prevention: the fear of judgment that often prevents high-risk individuals from engaging with health services. 

### Interpretation and mechanisms

Several mechanisms likely contributed to the observed improvements. First, the participatory design fostered genuine community ownership and engagement, moving beyond extractive research to collaborative problem-solving. This approach enhanced intervention relevance, cultural appropriateness, and alignment with community priorities—factors consistently associated with implementation success in community health interventions [28, 29]. Second, the multi-level intervention addressed individual, interpersonal, and community factors simultaneously through individual counseling, peer support, family engagement, and community resource mobilization [30]. This ecological approach aligns with health promotion principles recognizing that sustainable behavior change requires supportive environments, not just individual motivation.

Third, village health volunteers serving as peer facilitators likely enhanced intervention credibility, accessibility, and cultural resonance compared to facility-based programs led by professional healthcare providers. Trusted community members who share participants’ cultural context, language, and lived experiences can more effectively bridge health literacy gaps and address barriers to behavior change [29]. Fourth, the iterative refinement process enabled continuous adaptation to emerging challenges and community feedback, improving intervention feasibility and acceptability. This flexibility—inherent to PAR but often absent from standardized interventions—may be particularly important in resource-limited rural settings where unforeseen barriers commonly arise [31].

The significant health literacy improvements across all five domains suggest that the intervention successfully progressed participants through functional, interactive, and critical health literacy levels. Functional literacy (accessing and understanding basic health information) was addressed through culturally adapted educational materials and repeated exposure. Interactive literacy (social skills and capacity to act on information) was developed through facilitated group discussions, peer learning, and supported goal-setting. Critical literacy (analyzing information and using it to exert greater control over health) emerged through problem-solving activities, barrier identification, and community advocacy skills development [32]. This comprehensive approach distinguishes our intervention from information-only programs that target functional literacy alone.

### Implications for practice

#### Healthcare service delivery

Our findings have important implications for primary healthcare redesign in Thailand and similar LMIC settings. The successful community-based implementation suggests that diabetes prevention need not rely exclusively on facility-based clinical services, which face capacity constraints and accessibility barriers in rural areas. Instead, prevention can be effectively integrated into community structures using existing resources—health volunteers, temples, local organizations—with healthcare professionals assuming supportive rather than primary roles. This model aligns with Thailand’s Universal Health Coverage goals and the World Health Organization’s emphasis on community-centered primary healthcare.

Healthcare administrators and policymakers should consider: (1) formalizing partnerships between health facilities and community organizations for prevention programming; (2) investing in training and support for village health volunteers as frontline prevention agents; (3) adapting monitoring and evaluation systems to capture community-level prevention activities; and (4) allocating resources for participatory needs assessments and intervention co-design rather than implementing standardized programs. 

#### Health promotion programming

Health promotion practitioners can apply several lessons from this intervention. First, genuine community participation from initial planning through evaluation enhances intervention relevance and sustainability. This requires time investment, relationship-building, and willingness to cede control to communities—departures from traditional top-down programming. Second, interventions should leverage existing community capital rather than introducing entirely new structures, reducing resource requirements and facilitating post-study sustainability. Third, addressing stigma and creating supportive environments are as important as providing health information. Participants emphasized the importance of “close-up without serious risk”—being monitored and supported without judgment or alarmism—which enabled engagement without fear or shame.

Fourth, culturally adapting interventions beyond surface-level translation is essential. Our intervention incorporated local foods, agricultural schedules, traditional gathering spaces, communication preferences, and cultural values around family and community obligation. Finally, combining health literacy development with practical skill-building and environmental supports enables translation of knowledge into sustained behavior change.

### Implications for policy

#### Decentralization and local health governance

Thailand’s decentralization policy provides an enabling environment for community-based health promotion, but implementation gaps persist [12]. Our study demonstrates how subdistrict and provincial administrative organizations can effectively support diabetes prevention through resource allocation, inter-sectoral coordination, and community capacity-building. Policymakers should consider: (1) including community-based prevention metrics in local health performance indicators; (2) providing technical and financial support for participatory intervention development at the subdistrict level; (3) facilitating knowledge exchange among communities implementing prevention programs; and (4) ensuring that decentralization includes genuine authority and resources for local health decision-making, not just responsibility. 

#### National diabetes prevention strategy

Our findings support incorporating community-based participatory approaches into Thailand’s national diabetes prevention strategy. Current efforts focus predominantly on screening and risk identification, with less emphasis on comprehensive prevention programming for identified high-risk individuals [2]. Evidence-based, culturally appropriate prevention models like the one developed in this study could be adapted and scaled across rural communities facing similar challenges. This requires: (1) developing implementation toolkits and training programs to support community-led intervention adaptation; (2) establishing funding mechanisms for community prevention initiatives; (3) creating enabling policies that facilitate inter-sectoral collaboration; and (4) building evidence through continued implementation research and quality improvement. 

#### Cross-sectoral collaboration

Effective diabetes prevention requires collaboration beyond the health sector. Our intervention benefited from involvement of education (teachers), local governance (SAO, PAO), community leadership (village leaders), and volunteers. Policies enabling and incentivizing such collaboration—through joint planning processes, shared outcome accountability, and integrated resource allocation—could enhance prevention effectiveness and efficiency. This aligns with Health in All Policies approaches increasingly recognized as essential for addressing complex health challenges like diabetes.

### Strengths and limitations

This study has several notable strengths. First, the PAR methodology ensured that intervention development was genuinely community-driven, enhancing ecological validity and potential for sustained implementation. All four PAR phases were rigorously implemented with meaningful community participation throughout. Second, the mixed-methods approach provided complementary quantitative evidence of effectiveness and qualitative insights into mechanisms and contextual factors, yielding richer understanding than either method alone. Third, the diverse stakeholder involvement—including high-risk individuals, health volunteers, healthcare providers, local leaders, and administrators—enhanced intervention comprehensiveness and community-system integration. Fourth, the use of validated instruments with established validity and reliability may have reduced bias associated with self-reported measurements. In addition, electronic devices used to assess clinical outcomes were regularly checked and calibrated, which may have enhanced the accuracy and reliability of the clinical measurements. Lastly, methodological triangulation was employed to strengthen the rigor of the qualitative component. The research team consisted of investigators from diverse health-related disciplines with experience in qualitative research; data were collected through both in-depth interviews and focus group discussions (FGDs), and data analysis was conducted independently by multiple researchers.

Several limitations should be acknowledged. First, the single-group pre-post design without a control group limits comparison between this study and regular treatments. Observed improvements may partly reflect secular trends, regression to the mean, or measurement effects rather than intervention effects alone. In addition, seasonal events, life events, and Hawthorne effects might impact changes across multiple outcomes. Pragmatic and ethical considerations made randomization challenging in this small community where contamination between groups would be inevitable and withholding a community-desired intervention difficult to justify.

Second, the small sample size (*n* = 15) and single-site design limit statistical power and generalizability. The findings may not be generalizable to other settings, such as different regions or community structures. However, the pragmatic sample size was appropriate for intensive PAR methodology and pilot model development, and the detailed process description enables adaptation rather than direct replication in other contexts. This feasibility study warrants further investigation through a large-scale, multi-site effectiveness evaluation.

Third, the eight-week intervention duration, while sufficient to demonstrate significant changes, may be insufficient to assess long-term sustainability of behaviors and outcomes. Diabetes prevention requires sustained behavior change over years to decades. Extended follow-up is needed to determine whether improvements persist and whether participants maintain reduced diabetes risk. Fourth, the absence of objective dietary and physical activity measurement (e.g., accelerometry, dietary recall) limits precision in identifying specific behavior changes driving observed outcomes. Self-reported health records may be subject to social desirability bias and recall inaccuracy. Also, these are estimated parameters rather than gold standard measures.

Fifth, while we included diverse stakeholders, participation was voluntary, potentially selecting more motivated individuals. Findings may not represent experiences of less engaged community members. Finally, the researchers’ dual roles as intervention developers and evaluators introduce potential bias despite efforts to ensure rigorous data collection and analysis procedures. 

#### Directions for future research

Several research directions would advance understanding of community-based diabetes prevention. First, controlled effectiveness trials comparing participatory community-based interventions to usual care or facility-based programs would strengthen causal evidence and enable cost-effectiveness evaluation. Cluster randomized designs randomizing communities rather than individuals could address contamination concerns while enabling causal inference.

Second, long-term follow-up studies (12–24 months post-intervention) are needed to assess sustainability of health literacy gains, behavior changes, and clinical improvements. Understanding factors predicting sustained engagement versus relapse would inform maintenance strategies. Longitudinal studies should also examine actual diabetes incidence as the ultimate prevention outcome, which requires larger samples and extended observation.

Third, implementation research examining intervention adaptation, scale-up, and integration within routine healthcare delivery would inform translation from pilot to practice. Studies should explore resource requirements, implementation fidelity, reach across diverse populations, and sustainability under real-world conditions. Comparative effectiveness research examining different facilitation models (e.g., health volunteers versus community health workers versus peer leaders) would identify optimal approaches for various contexts.

Fourth, mechanism research investigating how participatory processes, peer support, family engagement, and other intervention components contribute to outcomes would enable intervention optimization. Mediation analyses could test hypothesized pathways from community participation through health literacy enhancement to behavior change and clinical improvement. Understanding why interventions work enables more efficient design. Fifth, research adapting and testing this model in different settings—urban communities, other LMIC countries, diverse cultural contexts, populations with different risk profiles—would assess transferability and identify necessary adaptations. Multi-site studies would enable evaluation of context-specific versus universal success factors.

Finally, research incorporating implementation science frameworks (e.g., RE-AIM, CFIR) systematically assesses reach, effectiveness, adoption, implementation, and maintenance dimensions, providing comprehensive evidence for scale-up decisions. Economic evaluations examining cost-effectiveness from societal, healthcare system, and participant perspectives would inform resource allocation and policy decisions.

## Conclusions

This participatory action research successfully developed and evaluated a community-based health literacy model that significantly improved health literacy and clinical outcomes among individuals at high risk for T2DM in rural northeastern Thailand. The intervention leveraged community capital, engaged diverse stakeholders, promoted self-management, and provided supportive monitoring to achieve meaningful improvements in preventive capacity. Four key success factors—community capital, healthcare reform thinking, self-initiated health management, and non-stigmatizing support—underpinned intervention effectiveness and offer insights for community-based prevention programming. The feasibility study demonstrates that intensive, resource-efficient diabetes prevention is feasible in rural LMIC settings through participatory approaches that engage communities as active partners rather than passive recipients. By integrating functional, interactive, and critical health literacy development within a supportive community environment, the intervention enabled participants to make informed decisions, enact health-promoting behaviors, and reduce T2DM risk. As Thailand and other LMICs confront rising diabetes prevalence amid constrained healthcare resources, innovative prevention models that maximize community capacity and minimize facility dependence warrant serious consideration. Future research should examine long-term sustainability, test effectiveness through controlled trials, explore implementation at scale, and investigate transferability across varied contexts. With continued development and evaluation, participatory community-based approaches hold substantial promises for reducing diabetes burden and advancing health equity in resource-limited settings globally.

## Data Availability

The datasets used and/or analyzed during the current study are available from the corresponding author on reasonable request.

## Abbreviations

BMI: Body mass index
CBPR: Community-based participatory research
FGD: Focus group discussion
LMIC: Low- and middle-income country
PAO: Provincial Administrative Organization
PAR: Participatory action research
SAO: Subdistrict Administrative Organization
T2DM: Type 2 diabetes mellitus
